# Chromosomal-level genome assembly of potato tuberworm, *Phthorimaea operculella*: a pest of solanaceous crops

**DOI:** 10.1038/s41597-022-01859-5

**Published:** 2022-12-03

**Authors:** Mengdi Zhang, Xinyue Cheng, Runmao Lin, Bingyan Xie, Ralf Nauen, Silvia I. Rondon, Jorge A. Zavala, Subba Reddy Palli, Suhua Li, Xingyao Xiong, Wenwu Zhou, Yulin Gao

**Affiliations:** 1 State Key Laboratory for Biology of Plant Diseases and Insect Pests, Institute of Plant Protection, Chinese Academy of Agricultural Sciences, Beijing, China; 2 College of Life Sciences, Beijing Normal University, Beijing, China; 3 Institute of Vegetables and Flowers, Chinese Academy of Agricultural Sciences, Beijing, China; 4 Key Laboratory of Green Prevention and Control of Tropical Plant Diseases and Pests Ministry of Education, College of Plant Protection, Hainan University, Haikou, China; 5 Bayer AG, Crop Science Division, R&D, Monheim, Germany; 6 Oregon State University, Hermiston Agricultural Research and Extension Center, Hermiston, OR USA; 7 Consejo Nacional de Investigaciones Científcas y Técnicas/Instituto de Investigaciones en Biociencias Agrícolas y Ambientales, Facultad de Agronomía, Universidad de Buenos Aires, Avda. San Martín, C1417DSE Buenos Aires, Argentina; 8 Department of Entomology, University of Kentucky, Lexington, Kentucky USA; 9 Agricultural Genomics Institute at Shenzhen, Chinese Academy of Agricultural Sciences, Shenzhen, Guangdong China; 10 State Key Laboratory of Rice Biology & Ministry of Agricultural and Rural Affairs Key Laboratory of Molecular Biology of Crop Pathogens and Insect Pests, Institute of Insect Sciences, Zhejiang University, Hangzhou, China; 11 National Center of Excellence for Tuber and Root Crop Research, Chinese Academy of Agricultural Sciences, Beijing, China

**Keywords:** Genome, Entomology

## Abstract

The potato tuberworm, *Phthorimaea operculella* Zeller, is an oligophagous pest feeding on crops mainly belonging to the family Solanaceae. It is one of the most destructive pests of potato worldwide and attacks foliage and tubers in the field and in storage. However, the lack of a high-quality reference genome has hindered the association of phenotypic traits with their genetic basis. Here, we report on the genome assembly of *P*. *operculella* at the chromosomal level. Using Illumina, Nanopore and Hi-C sequencing, a 648.2 Mb genome was generated from 665 contigs, with an N50 length of 3.2 Mb, and 92.0% (596/648.2 Mb) of the assembly was anchored to 29 chromosomes. In total, 16619 genes were annotated, and 92.4% of BUSCO genes were fully represented. The chromosome-level genome of *P*. *operculella* will provide a significant resource for understanding the genetic basis for the biological study of this insect, and for promoting the integrative management of this pest in future.

## Abstract (summary)

**Table:** 

Measurement(s)	whole genome sequencing
Technology Type(s)	Illumina NovaSeq. 6000 • Oxford Nanopore Sequencing
Sample Characteristic - Organism	Phthorimaea operculella
Sample Characteristic - Location	Yunnan Province, Southwest China

## Background & Summary

**Fig. 1:**
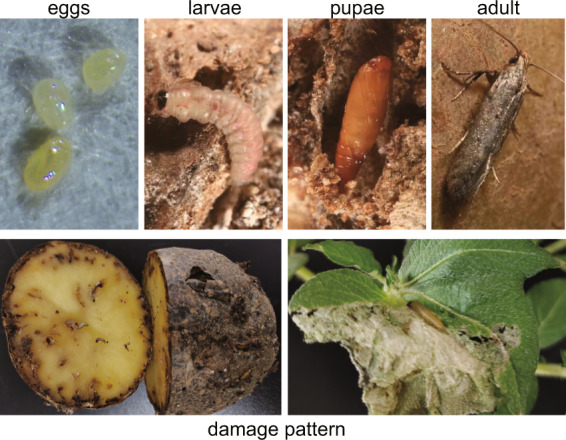
The potato tuberworm *Phthorimaea operculella* and its damage on potato plant.

The potato tuberworm, *Phthorimaea operculella* Zeller (Lepidoptera: Gelechiidae), is one of the main pests affecting potatoes, *Solanum tuberosum*, worldwide (Fig. 1). As an oligophagous pest of plants in the family Solanaceae, it uses potato, tomato (*S. lycopersicum*), and tobacco (*Nicotiana tabacum*) as principal hosts. It was first described in California in 1856. Since then, its presence has been reported in over 90 countries^1^. *Phthorimaea operculella* larvae feed on potato leaves, stems and petioles in the field, and tubers in storage. Severe infestations can destroy the foliage and results in substantial yield loss; however, main damage is the one that affects tubers. For instance, in some developing countries, the larvae can cause a 50–90% economic loss in storage; within weeks, tubers can become unmarketable if left untreated^2^. Pesticide application is most widely used management strategy to control *P. operculella*. Unfortunately, it can cause the development of insecticide resistance and negatively impact agro-ecosystem^3,4^. Thus, to promote more innovative management strategies for this destructive pest, a deeper understanding of its genetics is required but remains to be accomplished.

*P. operculella* belongs to the family Gelechiidae which is one of the most diverse families of microlepidoptera. Gelechiidae includes over 4700 described species in more than 500 genera in the world^5^. Many species of this family are considered important agricultural pests and feed voraciously on Solanaceous crops. *P*. *operculella*, the Guatemalan potato tuber moth *Tecia solanivora* Povolny, the tomato leaf miner *Tuta absoluta* Meyrick, the tomato pinworm *Keiferia lycopersicella* Walsingham, etc are among the major pests of this family. The genomic information for this family, however, remains scarce. Tabuloc *et al*. recently constructed a draft genome assembly for *T*. *absoluta*; the group also sequenced a preliminary genome of *K*. *lycopersicella* and *P*. *operculella*, with which a panel of 21-SNP markers^6^. Accumulating genomic information in the Gelechiidae family could promote a better understanding of the supra-specific classification within species^7^. To promote future studies on the genetics, biology, and ecology of Gelechiidae, it is of importance to build a chromosomal-level high quality genome assembly for important species such as *P*. *operculella*.

In the current study, we present a high-quality *P*. *operculella* chromosome-level genome assembly and life cycle transcriptomes. Using Illumina short reads, Nanopore, and High-throughput chromosome conformation capture (Hi-C) data, a 648.2 Mb genome was generated from 665 contigs, with an N50 length of 3.2 Mb, and 92.0% (596/648.2 Mb) of the assembly was anchored to 29 chromosomes. The female-specific W chromosome of P. operculella was not dertermined in this genome, since the identification of W chromosome is challenging due to high degeneracy, being gene-poor and repeat-rich^8^. In total, 16441 genes were annotated. Our genomic features of *P*. *operculella* will lay a foundation for further research on this insect pest.

## Methods

### Sample collection and sequencing

In 2014, *P. operculella* adults (n = 500) were collected from a potato field in Yunnan Province, China. The insect colony was maintained in the climate chamber at 27 ± 2°C, 60% RH and photoperiod of 12 h L: 12 h D. As in 2022, the colony has 100 generations of *P. operculella*. The chromosomal sex determination of *P. operculella* takes the form of female heterogamety (females are WZ, males ZZ)^9^. The male genome of *P. operculella* was thus sequenced to avoid the complications expected from the W chromosome of Lepidoptera^10^. DNA for both Illumina and Oxford Nanopore sequencing was obtained from 16 male pupae to avoid the contamination of eggs, and for Hi-C sequencing it was obtained from 200 mg fresh eggs.

The high-quality genomic DNA of *P. operculella* was prepared by the CTAB method and purified with QIAGEN® Genomic kit (QIAGEN, USA) at Grandomic Biosciences Co., Ltd (Wuhan, China), which was used for preparing the Illumina and Oxford Nanopore (ONT) sequencing libraries. The Illumina NovaSeq 6000 platform generated ~61 Gb of data with 150 bp paired-end reads, with an average insert size of 300~500 bp (Table 1). The Nanopore PromethION 24 platform generated ~68 Gb of sequencing data, and the adapters were removed using Porechop (https://github.com/rrwick/Porechop). The Hi-C library was constructed at Annoroad Gene Technology Co., Ltd (Beijing) following the standard library preparation protocol, and ~101 Gb of data with 50 bp paired-end sequencing raw reads were generated.

**Table 1:** Summary of sequencing data of *Phthorimaea operculella* genome.

Platforms	Nanopore (bp)	Illumina (bp)	Hi-c (bp)
Sum	68,640,761,997	61,377,501,600	101,236,335,300
Coverage	~108–123 x	~97–110 x	~159–180 x

Note: Platforms of Nanopore PromethION 24 and Illumina NovaSeq 6000 for reads sequencing.

With the Illumina sequencing data, we estimated the *P. operculella* genome size of ~636 Mb directly from kmer coverage from jellyfish v 2.0.0 analysis^11^; meanwhile, we used the Genomescope v1.0.0 method^12^ and estimated the genome size of ~560 Mb. The result suggested that the size of *P. operculella* may range from 560 to 636 Mb.

### RNA sequencing and analysis

Newly laid eggs, 1^st^, 2^nd^, 3^rd^ and 4^th^ instar larvae, mature larvae, pupae, and newly emerged adult moths were collected for transcriptome sequencing and gene expression analysis. Total RNA was isolated from eggs, larvae, and adults samples collected above, using Trizol reagent (Invitrogen, USA) following the manufacturer’s protocol. Illumina sequencing and complementary DNA (cDNA) library construction were performed at Grandomic Biosciences Co., Ltd (Wuhan, China). Clean data were obtained by removing adapters, low-quality reads, and high-content unknown sequences. Clean reads from each sample were mapped to the genome assembly to measure gene transcript levels using the reported analysis pipeline^13^.

### *De novo* genome assembly

**Fig. 2:**
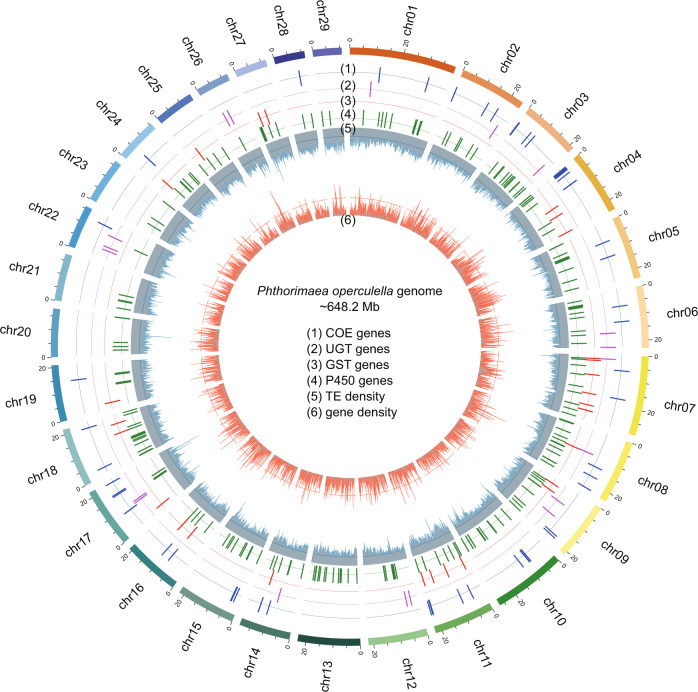
Characterization of the *Phthorimaea operculella* genome. Circos plot of chromosome level genome assembly (~648.2 Mb) and the distribution of COE, UGT, GST and P450 genes on 29 chromosomes.

**Fig. 3:**
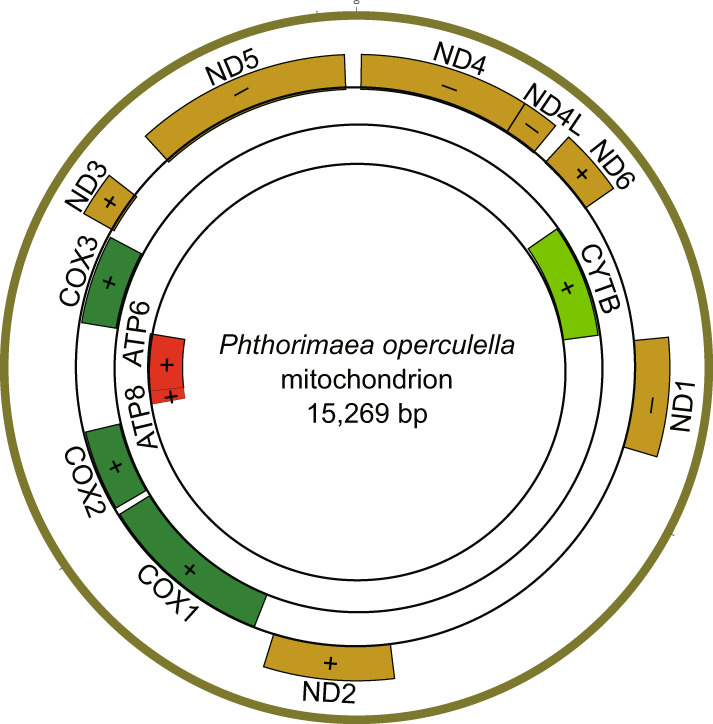
The assembly of the complete mitogenome (15,269 bp) of *Phthorimaea operculella*. 13 protein-coding genes (*ND1*-*ND6*, *ND4L*, *COX1*-*COX3*, *CYTB*, *ATP6* and *ATP8*) identified in the mitogenome were marked by coloured boxes.

Nanopore sequenced reads with a length of at least 8 kb were used for genome assembly by the Canu v1.8^14^ with parameters of “maxThreads = 60 genomeSize = 636 m -nanopore-raw”. For the primary assembly, the purge_dups^15^ was used to remove haplotypic duplication sequences, and Pilon^16^ and Racon^17^ were used to polish the assembly. Bacterial sequences that were identified by aligning against the NCBI nt database were also removed. After removing the mitogenome and bacterial sequences, we obtained the 665 contigs with size of 648.2 Mb, which was similar to the predicted size of ~560–636 Mb. The contig N50 size was 3.2 Mb. The analysis of Hi-C data helped to anchor 337 (50.7%) contigs of 596.3 (92.0%) Mb sequence to 29 chromosomes^18^ (Table 1 and Fig. 2). The 328 (49.3%) un-anchored scaffolds contained 51.9 (8.0%) Mb sequence. The mitochondrial genome of 15,267 bp was also obtained (Table 2 and Fig. 3).

**Table 2:** Statistics of genome assembly.

Genome assembly	Nuclear Genome	Mitogenome
Estimated size	~560−636 Mb	—
Chromosome	29	1
Contig size	648.2 Mb	15,269 bp
Contig number	665	1
Contig N50	3.2 Mb	15,269 bp
Longest contig	20.6 Mb	15,269 bp
GC content (%)	39.5	19.4
Gene number	16,619	13
Complete BUSCO (%)	92.4	—

### Repeat annotation

Transposable elements (TE), low complexity sequences and simple repeats were identified by RepeatMasker open-4.0.5 (http://www.repeatmasker.org) and RepeatScout^19^. Firstly, we used RepeatMasker to analyze low complexity sequences and simple repeats, as well as reference based TEs based on Repbase sequences v19.06^20^. Then we used the *de novo* method to discover TE families by running RepeatScout analysis, and these TE families were used for repeat annotation by running RepeatMasker analysis. In the 648.2 Mb genome assembly, 55.0% was repeat sequences^21^, including 54.2% of transposable elements (TEs), and 0.8% of simple repeats and low complexity sequences^22^.

### Protein-coding genes prediction and other annotation of the genome

Based on the genome sequence, we used Augustus^23^ and Genemark^24^ for ab initio gene prediction. And based on evidence from RNA-seq alignments and NCBI refseq invertebrate homology (https://ftp.ncbi.nlm.nih.gov/refseq/release/invertebrate/), we used Braker^25^ to infer gene models under three rounds of prediction (i.e., Braker + RNA-seq, Braker + refseq, Braker + RNA-seq + refseq). Then, we assigned priority to five gene sets (i.e., Braker + RNA-seq + refseq > Braker + RNA-seq > Braker + refseq > Genemark > Augustus), and selected genes supported by at least two methods or genes supported by only one method but containing functional domains. Moreover, all the selected genes may have similarity to reported invertebrate proteins or have RNA-seq evidence. Finally, we obtained 16,619 predicted protein-coding genes.

For five genomes of *Samia ricini*, *Dendrolimus punctatus*, *Drepana arcuata*, *T. absoluta* and *Carposina sasakii* without gene sets available at NCBI, we performed gene prediction analysis based on genome sequences using Augustus, Genemark and Braker + refseq methods, similar to those for the prediction of *P. operculella* genes. A total of 14,015, 14,483, 13,387, 17,607 and 15,873 genes were identified for *S. ricini*, *D. punctatus*, *D. arcuata*, *T. absoluta* and *C. sasakii*, respectively^26^.

**Fig. 4:**
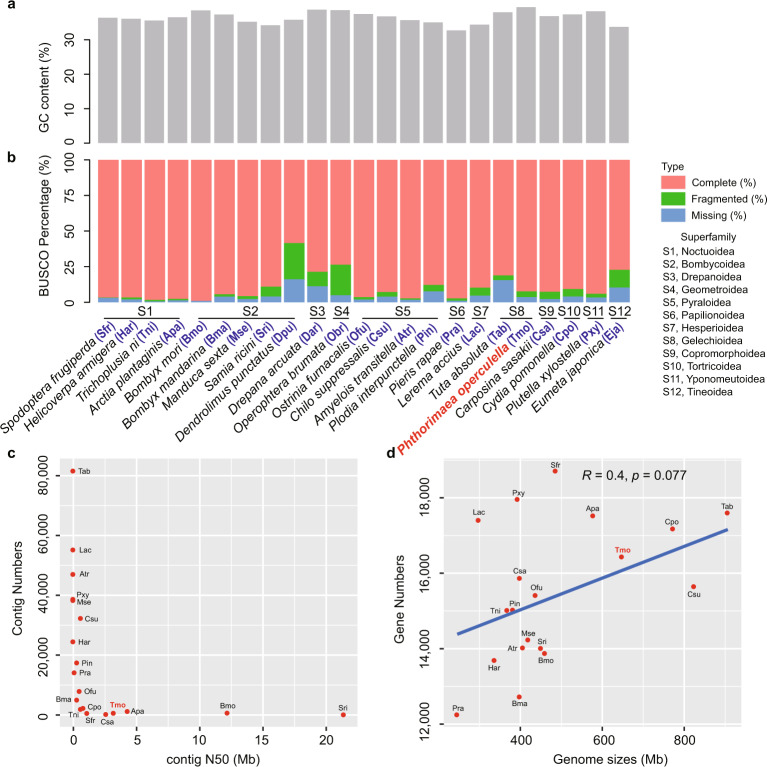
Comparison of characteristics between *Phthorimaea operculella* and 22 other Lepidopteran genomes. (**a**) GC contents of 23 lepidopteran genomes including species of 12 superfamily. (**b**) BUSCO scores for 23 assembled lepidopteran genomes. (**c**) Relationships between Contig N50 sizes and Contig numbers for 19 lepidopteran genomes with complete BUSCO scores above 80%. (**d**) Relationships between genome sizes and gene numbers for 19 lepidopteran genomes with complete BUSCO scores above 80%. The abbreviations for the name of each species were marked with blue.

For the amino acids of gene sets from 23 genomes within Lepidoptera and *L. decemlineata* genome within Coleoptera^22^, we used Benchmarking Universal Single-Copy Orthologs (BUSCO) v3.0.1^27^ to evaluate their quality. The “eukaryota_odb9” dataset at the BUSCO website (https://busco-archive.ezlab.org/v3/) was downloaded for analysis. The BUSCO completeness of >90% and >80% were found for gene sets from 16 and 20 genomes, respectively (Fig. 4).

**Fig. 5:**
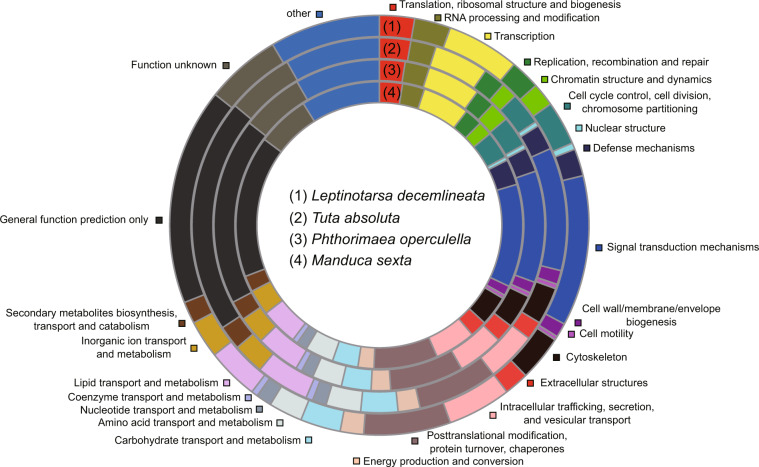
KOG annotations of four Solanaceae insect pests.

**Fig. 6:**
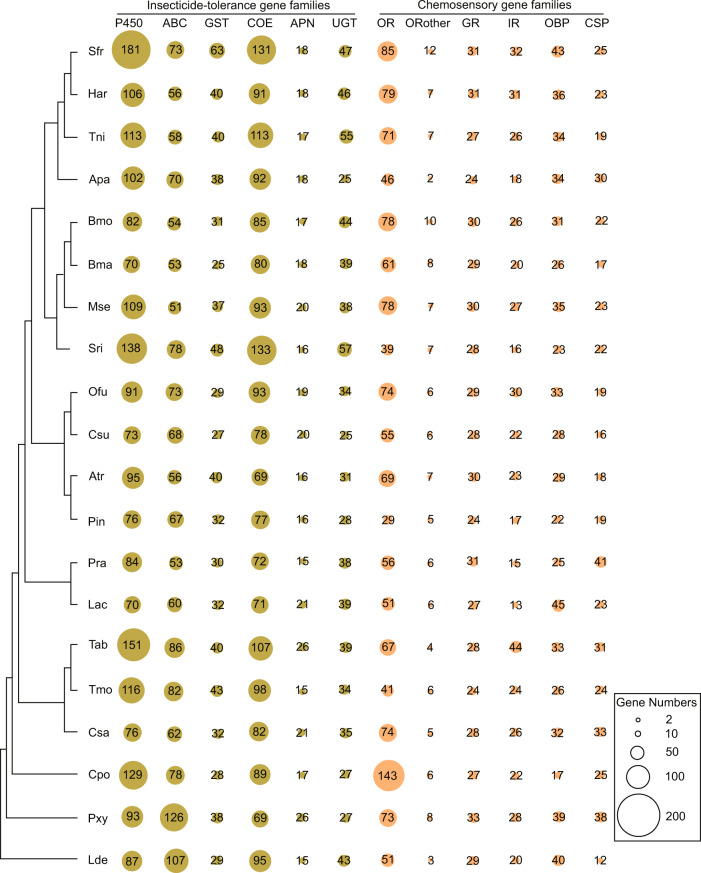
Distribution of detoxification and chemosensory genes in Lepidoptera species. P450, cytochrome P450 monooxygenase; ABC, ATP-binding cassette transporter; GST, glutathione S-transferase; COE, carboxylesterase; APN, aminopeptidase N; UGT, uridine diphosphate-glycosyltransferase; OR, olfactory receptor; GR, gustatory receptor; IR, ionotropic receptor; OBP, odorant-binding protein; CSP, chemosensory proteins.

To perform functional annotation, we aligned gene sequences against Pfam^22,28^, NCBI refseq invertebrate (https://ftp.ncbi.nlm.nih.gov/refseq/release/invertebrate/), UniProt^29^ and KOG^30^ databases using BLASTP with E-value cutoff of 1e-5 (Fig. 5). And pathway annotation was analyzed by KAAS^31^ online database server^22^. The P450 genes were annotated by aligning amino acids of genes against the collected data on Cytochrome P450 database (http://www.p450.unizulu.ac.za/)^32^. The genes from four sub-families (mito, CYP2, CYP3 and CYP4) were confirmed according to their annotation and orthogroup information (as described in the “Comparative genomic analysis”). The ABC transporters were identified based on UniProt annotation and orthogroup information of genes. All other metabolic enzyme genes were annotated based on domain annotations. The chemosensory genes containing the odorant receptor, olfactory receptor, the chemosensory receptor, PBP/GOBP family, and insect pheromone-binding family domains were annotated as OR, ORother, GR, OBP, and CSP genes, respectively. Genes of ligand-gated ion channels were annotated as IR genes. The sub-families (delta, epsilon, sigma, zeta, omega, theta and unclassified) of GST genes were annotated by comparing sequences against GSTs of *Plutella xylostella*^33^. These metabolic enzyme genes and chemosensory genes from 20 lepidopteran genomes with BUSCO completeness of larger than 80% were annotated^22^ (Fig. 6).

### Comparative genomic analysis for lepidopteran species

Twenty-four genomes were used for performing a comparative genomic analysis^22^, including 23 Lepidoptera genomes and one Coleoptera genome (*L. decemlineata*). These Lepidoptera genomes were from 17 families, with *T. absoluta* and *P. operculella* from the Gelechiidae family. The OrthoFinder v2.3.11^34^ detected 69,067 orthogroups for genes from these 24 genomes^22^, including 85 single-copy gene groups. Each orthogroup was considered as one gene family in the following analysis.

**Fig. 7:**
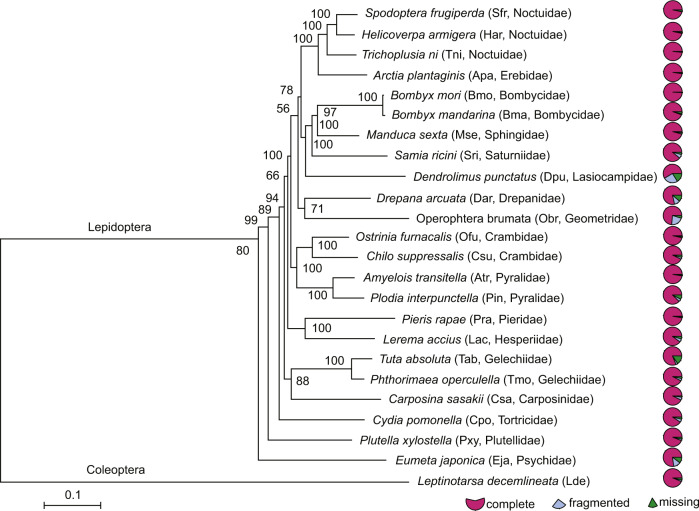
Phylogenetic analysis of 23 species in Lepidoptera. The best model of JTT + I + F + G with bootstrap value of 1000 replicates was used for constructing the phylogeny. The *Leptinotarsa decemlineata* from Coleoptera was used as outgroup.

**Fig. 8:**
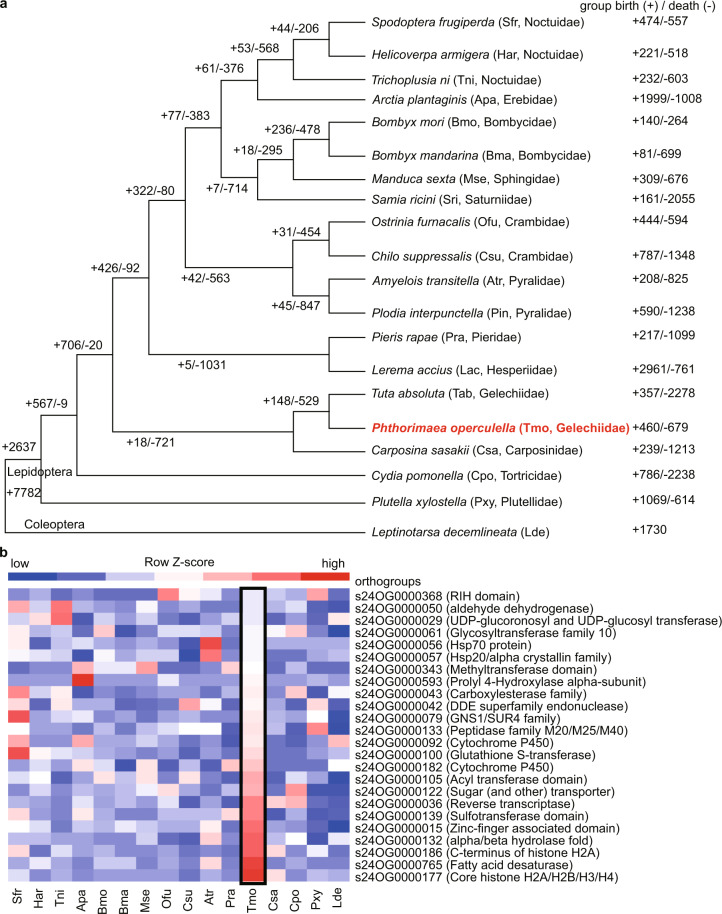
Gene family changes among lepidopteran insects. (**a**) Gene family changes associated with the origin and evolution of Lepidoptera. The topology of a phylogenetic tree constructed of 19 lepidopteran species. *Leptinotarsa decemlineata* (Coleoptera) was used as an outgroup. Gene family birth (+) and death (−) in 20 species are shown. (**b**) Expansion gene families in *P. operculella*, compared to other lepidopteran insects and *L. decemlineata*.

**Fig. 9:**
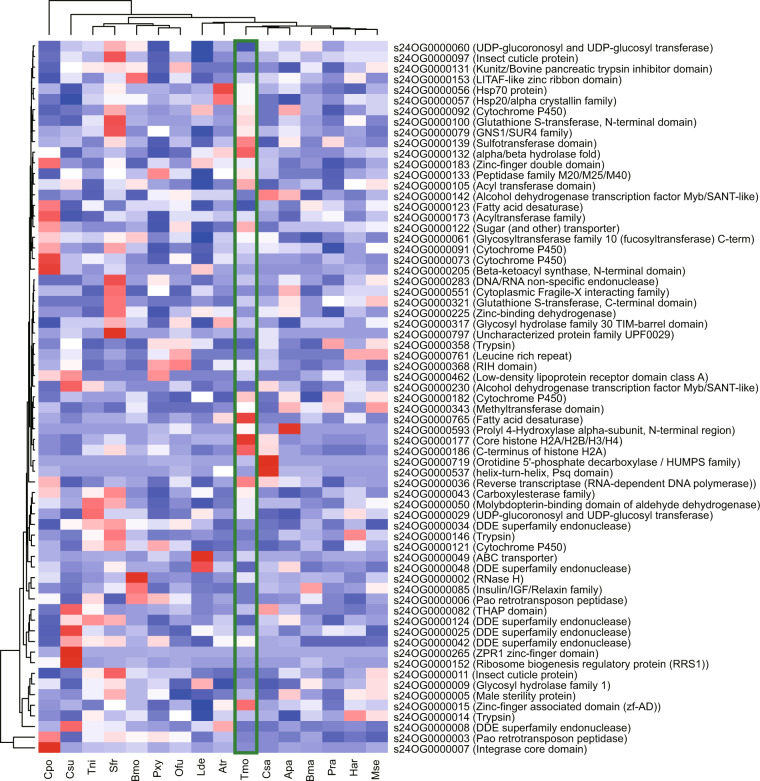
Expansion and contraction of gene families in 16 species. 67 gene orthogroups were found. The group-wide P-value of ≤ 0.01 was identified by CAFÉ analysis. The BUSCO complete of 90% was found for gene sets from these 16 species.

For each single-copy gene, we used MUSCLE v3.8.31^35^ to perform sequence alignment of amino acids. All the aligned genes were assembled by an in-house perl script (global_alignment_single_copy_genes.pl; https://github.com/linrm2010/global_alignment_single_copy_genes/). Then Gblock v0.91b^36^ was used to remove ambiguously aligned regions. The ProtTest v3.4^37^ identified the best model of JTT + I + F + G for constructing the phylogenetic trees. We used RAxML^38^ to construct the maximum likelihood phylogenetic tree for the 24 genomes (Fig. 7). After that, we analyzed the potential gene family emergence extinction according to the description in a previous study^39^, and applied CAFE v3.1^40^ to examine the expansion and contraction of gene families across the phylogenetic tree of genomes (Figs. 8,9).

## Data Records

The genome sequence and gene sequence had been deposited at the National Center for Biotechnology Information (NCBI), under the accession number of JANFCV000000000.1, and can be download from (ftp.ncbi.nlm.nih.gov/genomes/all/GCA/024/500/475/GCA_024500475.1_ASM2450047v1/)^41^. The NCBI BioProject accession number is PRJNA848272. The raw data of Nanopore, Illumina and Hi-C sequencing were submitted to NCBI SRA with the accession number of SRP405340^42^.Meanwhile, the genome sequence and gene sequence were also publicly available in National Genomic Data Center (NGDC), under the accession number of GWHBJUP00000000 (nuclear genome) and GWHBJUO01000000 (mitogenome). The gene expression data were publicly available in NGDC, under the accession number of OMIX001281. All data were related to the BioProject PRJCA010352.

## Technical Validation

We assessed the quality of genome assembly in the following aspects: (i) We obtained the complete mitogenome sequence of *P. operculella*. (ii) We aligned the Illumina sequencing reads against the nuclear genome using BWA v 0.7.17-r1188^43^, and found that 99.41% reads matched to genome sequences. (iii) The Core Eukaryotic Genes Mapping Approach (CEGMA) defined 458 core eukaryotic genes, and 248 of them were the most highly-conserved core genes, which could be used to assess the completeness of the genome or annotations^44^. We aligned the *P. operculella* genes against these 248 core genes, and identified that 243 (97.98%) core genes have homologous genes in the *P. operculella* gene sets. (iv) The BUSCO^20^ analysis showed that 96.4% of gene orthologs were identified in *P. operculella*, including complete and fragment scores of 92.4% and 4.0%, respectively. These results showed that we obtained the high-quality genome of *P. operculella*.

## Data Availability

All software and pipelines used in this study were executed according to the manual and protocols of the published bioinformatic tools. The versions of software have been described in Methods. The parameters of software/programs are as follows: Jellyfish: count -C -m 21. Genomescope: Rscript genomescope-1.0.0/genomescope.R NGS_reads.histo 21 150 NGS_reads.genomescope. Canu: genomeSize = 636 m -nanopore-raw. minimap2: -x map-ont. purge_dups: −2 -T cutoffs -c PB.base.cov. BUSCO: -m prot -f -l eukaryota_odb9. ProtTest: -all-distributions -F -AIC -BIC. Default parameters were used for Porechop, Pilon, Racon, RepeatMasker, RepeatScout, RepeatScout, Augustus, Genemark, Prothint, Braker, hmmsearch, OrthoFinder, MUSCLE and Gblock.
